# Crosstalk between cancer stem cells and myeloid-derived suppressor cells: implications for tumor progression and immunotherapy

**DOI:** 10.3389/fimmu.2025.1691661

**Published:** 2025-11-24

**Authors:** Bo Wang, Xiaoguo Zhao, Shuxin Han, Yuekang Xu, Jinyao Li

**Affiliations:** 1 Xinjiang Key Laboratory of Biological Resources and Genetic Engineering, College of Life Science and Technology, Xinjiang University, Urumqi, Xinjiang, China; 2 Xinjiang Key Laboratory of Special Environment and Health Research, School of Public Health, Xinjiang Medical University, Urumqi, Xinjiang, China; 3 Animal Experimental Center, Xinjiang Medical University, Urumqi, Xinjiang, China

**Keywords:** myeloid-derived suppressor cells, cancer stem cells, tumor immune microenvironment, tumor progression, immunotherapy

## Abstract

Cancer stem cells (CSCs) represent a small subset of tumor cells populations characterized by their ability to self-renew and differentiate. These cells are often considered resistant to chemotherapy, radiotherapy, and immunotherapy, playing a crucial role in driving tumor progression and metastasis. To evade immune attacks, CSCs utilize various genetic and epigenetic strategies that diminish immune recognition, enhance tolerance to immune-induced cytotoxicity, and foster the development of a protective immunosuppressive microenvironment. This microenvironment is shaped by a group of key immunosuppressive cells, particularly myeloid-derived suppressor cells (MDSCs), which not only directly inhibit effector T cells and natural killer (NK) cells, facilitating the immune escape of CSCs, but also significantly contribute to the maintenance of tumor cell stemness and promote their metastasis. Conversely, the developmental signals of MDSCs are also regulated by CSCs. This complex interplay between MDSCs and CSCs adds layers of complexity to the cancer-immune cycle and the associated tumor treatment strategies. Therefore, understanding the detrimental interdependence between MDSCs and CSCs to effectively impede tumor progression has become heated topic in tumor immunology. In this review, we provide a timely summary of the latest studies on the reported characteristics of CSCs and MDSCs, discuss their interconnection during tumor progression, and evaluate various immunotherapeutic strategies targeting these cell populations.

## 1 Introduction

The hallmarks of cancer are conceptualized as the acquisition of a specific set of functional capabilities by human cells as they transition from normal to neoplastic growth states (1). These capabilities encompass sustaining proliferative signaling, evading growth suppressors, resisting cell death, inducing or accessing the vasculature, activating invasion and metastasis, reprogramming cellular metabolism, and avoiding immune destruction (1). The emergence of these malignant traits may be associated with a rare subset of cancer cells known as cancer stem cells (CSCs), which possess an unlimited capacity for self-renewal that drives tumorigenesis (2). The limited efficacy of conventional anticancer therapies, such as chemotherapy and radiotherapy, which predominant target the dynamic stages of cancer cells (e.g., the cell cycle and cell division), can be attributed to their high efficacy in eliminating only the larger non-CSC population within the tumor (3, 4). Consequently, CSCs become enriched following these treatments and subsequently initiate tumor metastasis (3). This underscores the critical need to monitor and elucidate the biological properties of CSCs within intratumoral heterogeneity, a key factor influencing the drug resistance of tumor cells (4, 5). Therefore, comprehending the characteristics of CSCs is essential for developing more effective treatment strategies to improve patient prognosis (6).

Similar to all living cell, the growth of CSCs relies heavily on its environment. Mounting evidence has shown that the fate of CSCs is influenced by both secreted factors and cell–cell interactions within the tumor microenvironment (TME), which is a highly structured ecosystem consisting of cancer cells surrounded by various nonmalignant cell types, all embedded within an altered, vascularized extracellular matrix (7, 8). This environment contains a range of dysregulated immune regulatory cells, particularly tumor-associated macrophages (TAMs), regulatory T cells (Tregs), type 2 natural killer T cells, and, most notably, myeloid-derived suppressor cells (MDSCs) (9, 10). MDSCs are a population of bone marrow (BM)-derived immature and heterogeneous cells that are activated and mobilized under pathological conditions, particularly in cancer (11). They inhibit anti-tumor immunity through multiple mechanisms, including the depletion of L-arginine in the TME via inducible nitric oxide synthase (iNOS) and arginase-1 (Arg-1), and the exhaustion of tryptophan through indoleamine 2,3-dioxygenase (IDO), which directly suppresses T cell activation (12–15). Additionally, MDSCs release reactive oxygen species (ROS) and nitric oxide (NO) to disrupt the T cell receptor (TCR)-CD8 complex, hindering antigen-specific T cell responses (14, 16). They also induce the expansion of Tregs and the polarization of TAMs toward the M2 phenotype, thereby forming a synergistic suppressive network. MDSCs are correlated with treatment resistance; clinical studies have demonstrated that high infiltration of MDSCs in cancer tissues is associated with poor patient prognosis and resistance to conventional therapies (17–19). Elevated levels of MDSCs in the circulating tumor microenvironment correlate with suboptimal clinical responses to immune checkpoint blockade (ICB), indicating that MDSCs may play a critical role in mediating resistance to these therapeutic approaches (15). Importantly, there exists a bidirectional dependence between MDSCs and CSCs. MDSCs maintain CSC stemness through pathways such as IL6/STAT3 and NO/NOTCH, while CSCs secrete factors such as granulocyte-macrophage colony-stimulating factor (GM-CSF), CXCL1, CXCL2, and CXCL8 to expand and recruit MDSCs (20–22). This intricate relationship complicates the cancer-immunity cycle and corresponding tumor treatment strategies. Therefore, it seems unlikely that a single strategy focused solely on MDSCs could produce a robust antitumor effect; the reciprocal influence of CSCs on MDSCs also needs to be considered. Based on this, we provide a timely summary of the latest studies on the main characteristics of CSCs and MDSCs, their mutual promoting effects during tumor development, and immunotherapies targeting MDSCs and CSCs.

## 2 CSCs

### 2.1 Origin

**Figure 1:**
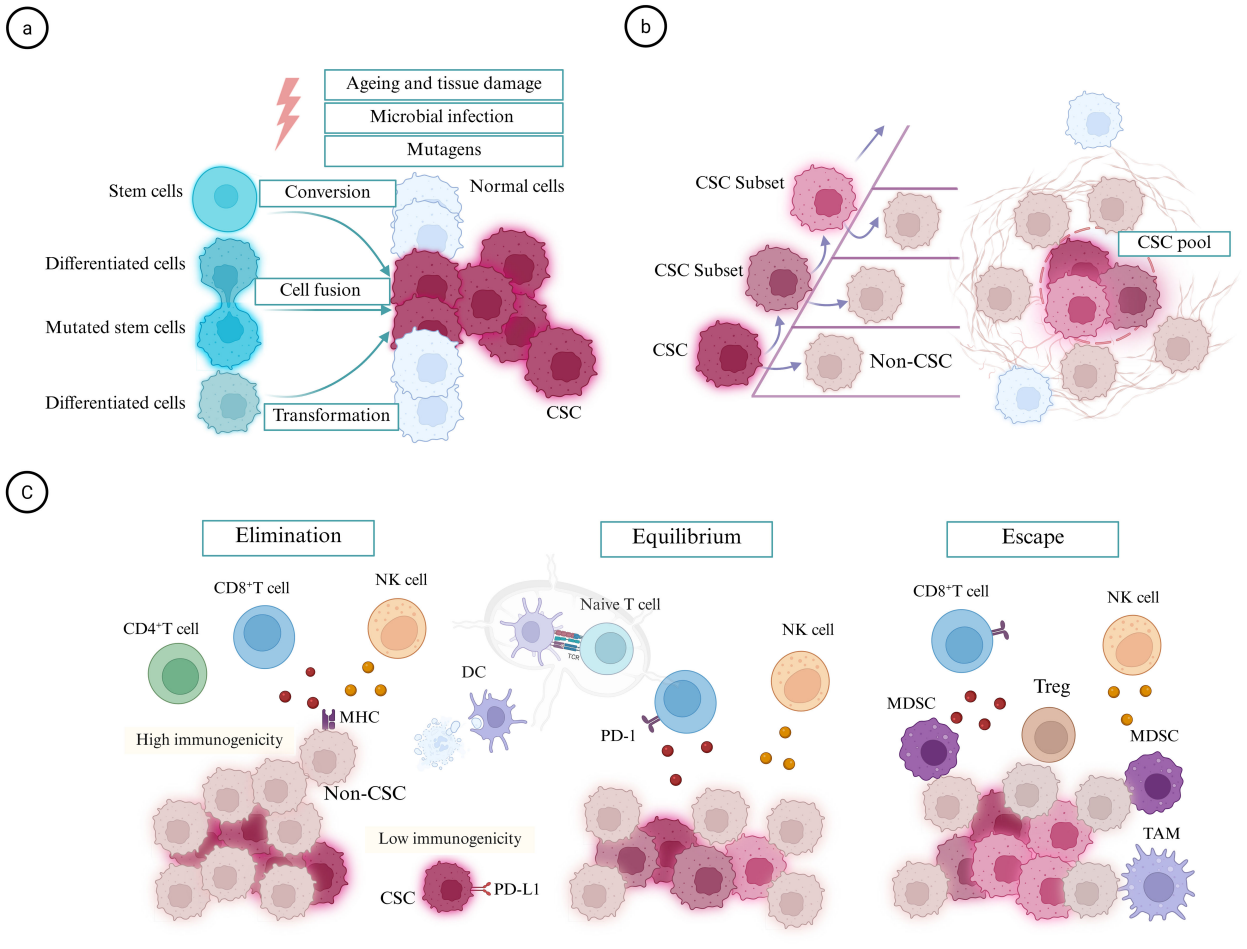
Origin and development of CSCs. **(a)** Under the influence of such carcinogenic factors as microbial infection, mutagens, chronic inflammation, and environmental stress, CSCs can arise from either transformation of stem cells, fusion with differentiated cells, or transformation of differentiated cells. **(b)** Once developed, CSCs initiate clonal expansion. Concurrently, CSCs may acquire various mutations that facilitate the formation of new subset, which in turn generate a substantial number of non-CSCs. Over time, these CSC subsets evolve and sustain their self-renewal capacity to resist damage depending on specific microenvironmental niches, ultimately resulting in the formation of a pool of CSCs. **(c)** Successful tumor development in the host involves three stages of battling with immune system. At early stages, tumor cells with high levels of immunogenicity are targeted by the immune system through immune surveillance, leading to their complete “elimination” if successful. Following the first round of immune destruction, tumor cells with moderate levels of immunogenicity may exist in a state of “equilibrium” with the immune system, resulting in the emergence of 'edited' tumor cells characterized by reduced antigen levels and increased inhibitory ligands (e.g., PD-L1), before the recruitment of immunosuppressive cells such as TAMs, Tregs, and MDSCs occurs to allow tumors to achieve immune “escape”. DC, dendritic cell; MHC, major histocompatibility complex; NK, natural killer; TAM, tumor-associated macrophage; Treg, regulatory T cell.

During the initial stages of tumorigenesis, CSCs can arise through various mechanisms, including the tumorigenic transformation of stem cells (4, 23), mutations in tumor suppressor genes induced by environmental stress (24), cell fusion between mutated stem cells and differentiated cells (25, 26), horizontal gene transfer between donor and recipient cells (26), and transdifferentiation from non-CSCs influenced by factors present in the TME (5). These mutated, fused, or transformed cells possess the ability to self-renew and differentiate, giving rise to both CSCs and non-CSCs *in vivo* (23) (**Figures 1a, b**). For instance, neural stem cells can be transformed through a combination of mutations, including the concurrent mutation of Nf1 and Pten alongside EGFRvIII overexpression, resulting in the formation of glioblastoma multiforme stem cells characterized by extremely aggressive tumor growth and infiltration *in vivo* (27). Furthermore, CSCs can be generated from stem cells or induced pluripotent stem cells without genetic manipulation when exposed to a cancer-inducing niche (28). An analysis of 146 independent human pluripotent stem cell line samples revealed that 22% of the samples contained at least one cancer-related mutation, primarily occurring during culture and reproduction (24). Moreover, differentiated cells are significantly more abundant than constitutively active adult stem cells. Due to their long lifespan and error-prone damage repair mechanisms, differentiated cells can undergo plasticity events, converting into stem-like cells, which represent a severely underestimated source of cancer (29).

### 2.2 Biomarkers

CSCs rely on specific intracellular and extracellular signals, as well as transcriptional programs to maintain their stemness, resulting in protein or epigenetic differences when compared to non-CSCs (30). While common biomarkers such as CD133, CD44, EpCAM, and ALDH are identified in CSCs across various tumor types, the heterogeneity of cancers often necessitates the utilization of multiple markers or a combination of intracellular and extracellular markers for the isolation and characterization of CSCs (31). For instance, in breast cancer, markers such as CD44^high^/EpCAM^high^, CD44^+^CD24^−/low^, CD49f^+^/EpCAM^+^, and CD49f ^high^/CD61^high^, as well as the combination of CD44^+^CD24^−/low^ and ALDH^+^, are employed to identify and isolate breast CSCs (32, 33) Additionally, the cell surface marker ABCG2, a member of the ATP-binding cassette superfamily G, can be utilized to select for CSCs (34). ABCG2^+^tumor cells may represent a distinct CSC population with inherent resistance to common antitumor drugs, potentially contributing to tumor recurrence (35). Activating intracellular pluripotency factors such as SRY-box transcription factor 2 (Sox2), Nanog homeobox (Nanog), and POU class 5 homeobox (Oct4) is crucial for regulating stem cell properties and serve as typical CSC biomarkers (36). Furthermore, compared to non-CSCs, CSCs exhibit unique characteristics, such as self-renewal, proliferation, and differentiation, which involve alterations in various intracellular pathways, including the Wnt, Notch, Hedgehog, PI3K/Akt, JAK/STAT, and NF-κB pathways. These altered pathways can also be regarded as intracellular biomarkers for CSCs (37). Moreover, CSCs typically exhibit lower stiffness as a biological feature, whereas differentiated mature cancer cells are characterized by increased in cell stiffness (38, 39). Although cytotoxic T lymphocytes (CTLs) can effectively eliminate stiff, differentiated cancer cells, they are unable to target soft, regenerating CSCs (40).

### 2.3 Tumor development

One of the essential functions of the immune system is to safeguard the host through cancer immune surveillance and tumor-sculpting activities (41). Tumors that develop in a fully functional immune system generally exhibit lower immunogenicity compared to those arising in immunodeficient hosts. This discrepancy occurs because, under immune pressure, genetic alterations can transpire in tumor cells, leading to a phenomenon known as cancer immune editing, which encompasses elimination, equilibrium, and escape stages (41, 42).

Early tumor cells employ subtle immune evasion strategies for survival, such as downregulating major histocompatibility complex (MHC) expression to present less immunogenic antigens while maintaining sufficient surface MHC levels to evade clearance by NK cells (43). This results in the emergence of less immunogenic tumor cell subclones. In a Kras-Trp53 driven lung adenocarcinoma mouse model, continuous and comprehensive monitoring of the entire process of a single cell carrying oncogenic mutations evolving into an invasive tumor was conducted based on single-cell RNA sequencing. This investigation revealed that rare subclones can drive tumor expansion through unique transcriptional programs (44). Additionally, these rare subclonal populations can survive during the immune clearance phase, potentially exhibiting characteristics associated with CSCs. The low immunogenicity of CSCs, coupled with defects in antigen processing and presentation, can suppress T cell proliferation, thereby demonstrating features of immune evasion (45). CSCs exhibit reduced surface expression of MHC-new antigen peptide presenting molecules, which may lead to partial resistance against specific CD8^+^ T cells (46). Furthermore, there is a possibility of "not yet immunoedited" cancer cells that survive the initial elimination stage, which may resemble CSCs akin to pluripotent stem cells that resist immune pressure through their stem cell microenvironment (47).

In addition to the aforementioned passive evasion mechanisms, CSCs can induce CD8^+^ T cell exhaustion through direct intercellular interactions or by secreting exosomes (48, 49). The primary manifestation of immune exhaustion is characterized by a reduced secretion of effector cytokines from T cells and an increased expression of inhibitory receptors such as PD-1 and TIM3 (48). On one hand, CSCs express elevated levels of PD-L1 on their surface, which specifically binds to PD-1 on CD8^+^ T cells, thereby directly obstructing TCR-CD3 signaling and inhibiting the secretion of effector molecules such as IFN-γ and granzyme B (48, 50). This blockade results in T cell functional inactivation and exhaustion. On the other hand, the exosomes secreted by CSCs can carry molecules such as PD-L1 and Tenascin-C, which act on CD8^+^ T cells from a distance: exosomal PD-L1 binds to T cell PD-1, enhancing the exhausted phenotype, while Tenascin-C inhibits mTOR signaling pathway activity by binding to α5β1/αvβ6 integrins on the T cell surface, thereby reducing the synthesis of effector molecules (49).

Although tumor evolution can be directed toward less immunogenic phenotypes through immunoediting, immunogenic heterogeneity remains an inherent characteristic of tumor cells. Recent studies have shown that the RNA binding protein cold shock domain–containing protein E1 (CSDE1) in nascent tumorigenic cells promotes the dephosphorylation of transducer and activator of transcription by stabilizing T-cell protein tyrosine phosphatase (TCPTP), thereby influencing the immunogenicity of new tumorigenic cells (51). The H3K4 trimethylation of the CSDE1 locus is regulated by SET and MYN domain–containing 3 (SMYD3) (51). The immunogenicity of nascent tumorigenic cells may be modulated by the cellular microenvironment prior to being edited by the immune system (52). Long-lived CSCs facilitate an environment conducive to the accumulation of mutations, ultimately leading to the emergence of an immune escape phenotype (47) (**Figure 1c**). This suggests that maintaining growth and immune balance requires synergistic regulation between CSCs and microenvironmental signals; once this synergy is disrupted, the balance tilts toward tumor progression. Once tumor cells with immune evasion phenotypes successfully breach the initial immune elimination barrier, a dynamic equilibrium is established between tumor growth and immune elimination (47, 53). The maintenance of this balance is intricately linked to the adaptive evolution of CSCs.

In the research on glioblastoma multiforme, engineered glioblastoma multiforme stem cells acquire immune evasion capabilities following serial transplantation in immunocompetent hosts (27). Depletion of the CD8^+^ T-cell population results in accelerated tumor growth and a progressive increase in tumor penetrance (27). This finding confirms that tumors arising from glioblastoma multiforme stem cells and their derivatives do not undergo antigen loss; rather, they remain under significant pressure from CD8^+^T-cell-mediated clearance. Importantly, the inhibition of colony-stimulating factor 1 receptor (CSF-1R) signaling reduces the ability of glioblastoma multiforme stem cells to evade immune responses (27). This indicates that maintaining a balance between growth and immunity requires synergistic regulation between CSCs and microenvironmental signals; once this synergy is disrupted, the balance tips toward tumor progression.

Specifically, the disruption of the dynamic equilibrium between tumor growth and immune clearance is closely associated with numerous dysregulated immune regulatory cells within the TME. These cells play a crucial role in inducing and maintaining the stemness and chemoresistance of CSCs through various biochemical factors, including cytokines, membrane proteins, and non-coding RNAs present in extracellular vesicles (54–56). In particular, the cooperative action of immunosuppressive cells enables CSCs to evade immune surveillance and inhibit anti-tumor immune responses (9, 10). For instance, PMN-MDSCs enhance the stemness and growth of colorectal cancer cells via exosomal S100A9 (57). In addition to the direct regulation of immune cells, the functional heterogeneity of specific transcription factors significantly affects tumorigenesis, with the role of IRF8 being particularly representative, as its function varies completely depending on the type of tumor cells.

IRF8 exhibits functional heterogeneity during tumorigenesis, demonstrating varying roles across different tumor cell populations. This heterogeneity manifests primarily in two distinct ways: IRF8 promotes oncogenesis in CSCs while suppressing it in differentiated common cancer cells, operating through completely opposite mechanisms. In glioblastoma, glioblastoma stem cells activate the expression of IRF8 via epigenetic immune editing in response to immune attacks from the host (27). Subsequently, IRF8 regulates myeloid-related transcriptional programs, directly upregulating the promoter activity of the chemokine CCL2. The elevated expression of CCL2 further recruits tumor-associated macrophages, thereby constructing an immunosuppressive microenvironment around CSCs (27). Silencing IRF8 in glioblastoma stem cells reduces CCL2 expression, significantly weakening the immune evasion capability of CSCs and directly confirming the pro-oncogenic role of IRF8 in these cells (27). This abnormal activation serves as a survival mechanism evolved under immune selection pressure and represents an adaptive strategy employed under varying microenvironmental conditions. In stark contrast to its pro-cancer role in CSCs, IRF8 consistently functions as a tumor suppressor in differentiated and mature common cancer cells. In breast cancer cells, IRF8 directly binds to the β-catenin protein, thereby inhibiting its translocation into the nucleus (58). This inhibition ultimately results in a reduced proliferation rate and diminished migratory and invasive capabilities of breast cancer cells. When IRF8 is silenced due to methylation, the tumorigenicity of colon cancer cells is significantly enhanced (59). High levels of IRF8 in cancer patients are significantly associated with the infiltration of activated effector CD8^+^ T cells (60). Beyond its direct regulation of cell proliferation and apoptosis, IRF8 inhibits the activation of the IL6-JAK-STAT3 pathway in lung adenocarcinoma cells, reducing the differentiation of MDSCs and significantly weakening the immunosuppressive microenvironment, thereby indirectly suppressing the progression of lung adenocarcinoma (61). It is noteworthy that the regulation of IL6 mediated by IRF8 is only one of the ways in which CSCs influence the immune microenvironment. CSCs can also construct an immune evasion barrier by directly secreting immunosuppressive cytokines.

CSCs directly secrete various immunosuppressive cytokines, facilitating immune evasion through cytokine-mediated suppression (50). Notably, CSCs secrete elevated levels of transforming growth factor-beta (TGF-β), which induces naive T cells to differentiate into Tregs, thereby enhancing local immunosuppression (50, 62). Concurrently, TGF-β inhibits the proliferation of CD8^+^ T cells and reduces the expression of the NKG2D receptor on NK cells, diminishing their cytotoxic activity against CSCs (50). Furthermore, CSCs secrete IL-6, which activates the STAT3 signaling pathway, preserving their stemness characteristics while promoting the expansion of MDSCs (20, 63). Additionally, IL-6 directly suppresses the secretion of IFN-γ from CD8^+^ T cells, thereby reducing their anti-tumor efficacy (63). Certain CSCs, including glioma CSCs and breast cancer CSCs, also secrete IL-10, which further enhances the local immunosuppressive microenvironment, diminishes effector T cell infiltration, and inhibits their activation (50).

## 3 MDSCs

### 3.1 Classification

**Figure 2:**
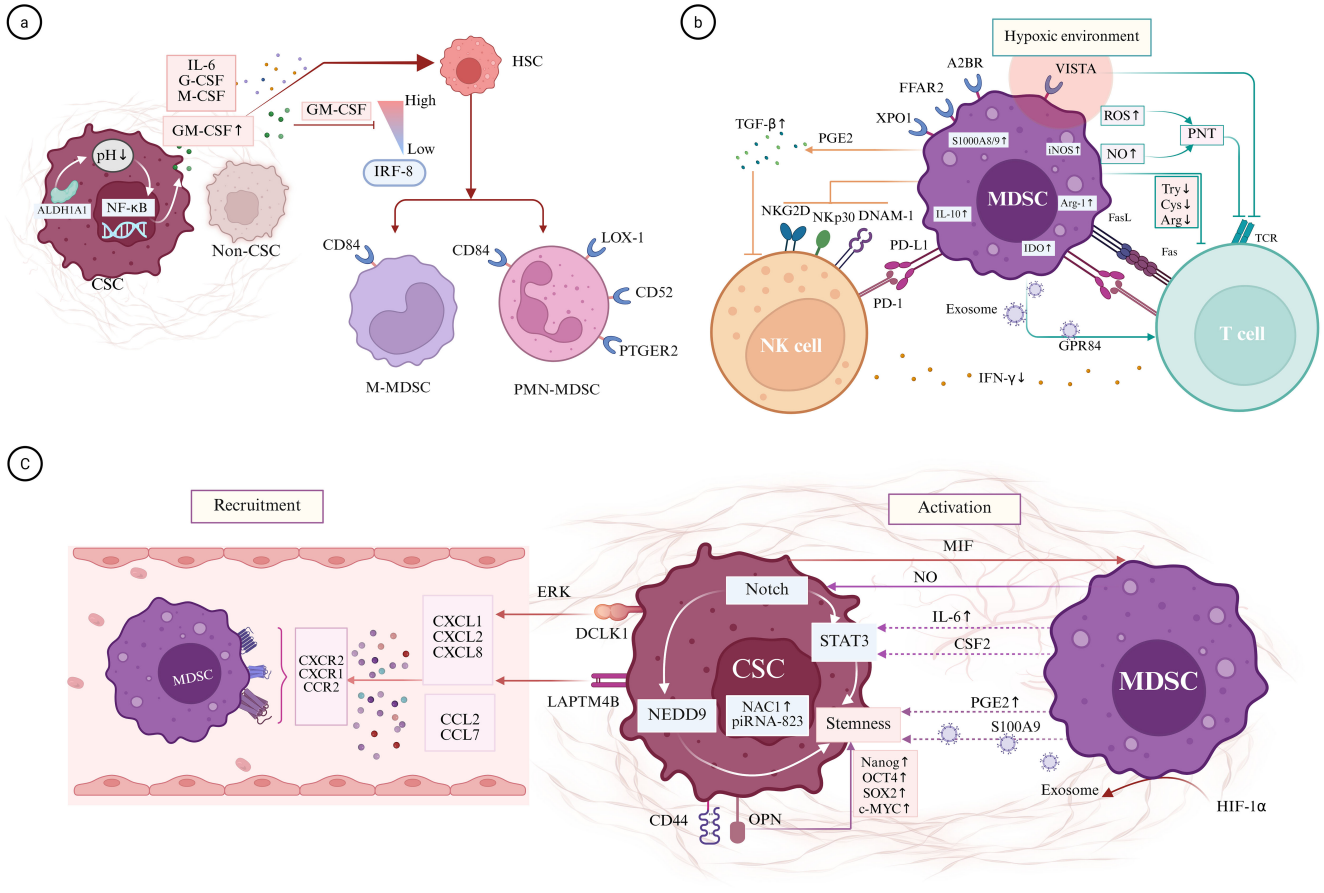
MDSCs and CSCs in cancer progression. **(a)** Development of MDSC and MDSC-related markers. The activity of ALDH1A1 in CSCs can lower the intracellular pH of cancer cells and activate NF-kB signaling to increase the secretion of GM-CSF and other tumor-derived signals, which impact HSCs, resulting in their suppressed expression of IRF8 to promote the differentiation of PMN-MDSCs and M-MDSCs. Potential markers of MDSCs include LOX-1, CD84, CD52, and PTGER2. **(b)** Immunosuppressive mechanisms of MDSCs. MDSCs inhibit T-cell function through receptor/ligand interaction and modulating essential metabolites such as arginine, tryptophan, and cysteine within the TME involving iNOS, Arg-1, and IDO. Furthermore, the expression of VISTA, XPO1, A2BR, FFAR2, and FATP2 on MDSCs is closely associated with the suppression of T cell activity. In addition, MDSCs also suppress NK cell cytotoxicity and IFN-γ production by downregulating NKp30, DNAM-1, and NKG2D while enhancing PGE2/TGF-β and PDL-1/PD-1 signaling. **(c)** The bidirectional interaction between MDSCs and CSCs within the TME. On one hand, MDSCs maintain the stemness of cancer cells through the IL6/STAT3, NO/NOTCH, NOTCH/NEDD9, and CSF2/p-STAT3 signaling pathways. Furthermore, they sustain the stemness of CSCs by expressing PGE2/S100A9/NAC1/piRNA-823. On the other hand, CSCs express DCLK1, LAPTM4B, and NEDD9, which regulate the expression of CXCL1, CXCL2, and CXCL8, thereby facilitating the recruitment of MDSCs into the TME. Additionally, CSCs express MIF to activate MDSCs. A2BR, adenosine receptor 2B; FFAR2, free fatty acid receptor 2; GM-CSF, granulocyte-macrophage colony-stimulating factor; HSC, hematopoietic stem cells; LOX-1, lectin-type oxidized LDL receptor-1; MMP, matrix metalloproteinases; PGE2, prostaglandin E2; PNT, Peroxynitrite; VISTA, V-domain Ig suppressor of T-cell activation; XPO1, exportin 1.

In addition to intrinsic factors, tumorigenesis is also facilitated by signals from extrinsic factors. As normal bronchial tissue progresses through various lesion stages, there is an increase in the total number of neutrophils and myeloid-derived cells, which coincides with the detection of elevated levels of cancer germline antigens associated with lung squamous cell carcinoma (64). These pathologically activated myeloid cells are classified as MDSCs, which primarily consist of immature myeloid cells, including polymorphonuclear cells from the granulocytic lineage (PMN-MDSCs) and monocytic cells (M-MDSCs) (65) (**Figure 2a**). They share several key biochemical characteristics that enable them to suppress immune responses, including the upregulation of STAT3 expression, as well as the expression of Arg-1 and S100A8/A (66). However, the mechanisms of immune response modulation differ; for instance, PMN-MDSCs primarily utilize reactive ROS, peroxynitrite (PNT), and Arg-1 to mediate immune suppression, whereas M-MDSCs achieve this through the production of NO and immunosuppressive cytokines (66). Notably, in tumor tissues, the M-MDSC population expands less than the PMN-MDSC population, and M-MDSCs rapidly differentiate into TAMs and inflammatory DCs (67).

Due to the phenotypic similarities between PMN-MDSCs and classical neutrophils, it is crucial to identify biomarkers that can effectively distinguish PMN-MDSCs from classical neutrophils, which are characterized by the expression of CD11b^+^Ly6G^+^Ly6C^-/low^. Transcriptome analysis of PMN-MDSCs and neutrophils from the same patient revealed unique gene profiles (68). The elevated expression of lectin-type oxidized LDL receptor-1 (LOX-1) in PMN-MDSCs serves as a distinguishing marker compared to neutrophils; however, it does not differentiate between immature and mature PMN-MDSCs (68, 69). PMN-MDSCs are characterized by a core of chronic immune suppression, with an enrichment of genes associated with immune suppression, such as Arg1 and iNOS, which impede antigen-specific T cell responses (12–14, 66, 68). Beyond serving as a phenotypic marker, LOX-1 also mediates the endoplasmic reticulum (ER) stress response, thereby maintaining the pathological activation state of PMN-MDSCs and ensuring sustained secretion of immune-suppressive molecules (68, 69). In contrast, neutrophils are oriented toward acute immune clearance, exhibiting an enrichment of genes related to pathogen recognition and direct killing, such as myeloperoxidase (MPO) and neutrophil elastase (NE), which facilitate rapid clearance through the production of hypochlorous acid and the degradation of pathogen cell walls or tumor stroma (12, 70). Neutrophil pattern recognition receptor-related genes, including TLR2, TLR4, and components of the NF-κB pathway, are highly expressed and can swiftly identify pathogen-associated molecular patterns (PAMPs) or damage-associated molecular patterns (DAMPs), thus activating acute inflammatory responses to initiate immune surveillance (12, 70). Consequently, the differential gene expression patterns between PMN-MDSCs and neutrophils dictate their functional roles in immune surveillance: neutrophils serve as acute immune defense units, rapidly clearing pathogens or early tumor cells through the high expression of genes related to killing, pathogen recognition, and glycolysis, thereby initiating effective immune surveillance. Conversely, PMN-MDSCs suppress the function of effector immune cells, such as T cells and NK cells, by enriching genes associated with immune suppression and ER stress, which disrupts immune surveillance and ultimately promotes tumor immune evasion.

Furthermore, CD84 has been identified as a surface marker for detecting and enriching MDSCs (69, 71), while CD300Id is essential for recruiting PMN-MDSCs into tumors and inhibiting T-cell activation (72). Similarly, CD52 and prostaglandin E receptor 2 (PTGER2) may serve as potential markers related to mature PMN-MDSCs (69) (**Figure 2a**). Notably, classical neutrophils exhibiting anti-tumor properties are the predominant responding cells during the early stages of tumor development (73). However, as the tumor progresses, a branch of the developmental pathway of classical neutrophils is activated, leading to the expansion and accumulation of PMN-MDSCs, which are subsequently recruited to the tumor microenvironment (66). This process contributes to the establishment of an immunosuppressive environment, thereby facilitating tumor evasion.

### 3.2 Dual signals for expansion and activation

The generation and activation of MDSCs is a dual-faceted process. One pathway primarily drives the expansion and accumulation of MDSCs, while the other contributes to their pathological activation (12, 15). These two distinct signal transduction pathways are continuously interconnected and are referred to as the dual signal model (12). MDSCs are predominantly generated in the BM, where tumor-derived factors influence the differentiation of BM cells. The activity of ALDH1A1 in tumor-initiating cells can lower the intracellular pH of breast cancer cells, promote the phosphorylation of TAK1, activate NF-kB signaling, and increase the secretion of GM-CSF (21). This cascade ultimately leads to the expansion, activation, and acquisition of immunosuppressive properties by MDSCs, thereby facilitating the progression of breast cancer (21). Furthermore, various signaling factors influencing the expansion of MDSC include G-CSF, M-CSF, S-SCF, VEGF, and polyunsaturated fatty acids, which affect the expression and function of transcription factors involved in BM differentiation (12). Among these transcription factors, IRF8 acts as a negative regulator of MDSC biology (61, 74). The overexpression of IRF8 has been shown to inhibit the growth of tumor-induced MDSCs in mouse models, while IRF8 deficiency facilitates granulocyte expansion (74). Additionally, the loss of SHP-2 enhances the GM-CSF-mediated phosphorylation of IRF8, thereby influencing MDSC differentiation (75). In comparison to normal colon tissue, human colorectal cancer tissues exhibit significantly elevated levels of DNMT1 and DNMT3b, reduced expression of IRF8, and increased DNA methylation at the IRF8 promoter (59). T cells lacking in IRF8 demonstrate increased expression and secretion of GM-CSF, resulting in dysregulated myeloid differentiation and MDSC expansion in corresponding mice (76). Other notable transcription factors and regulators involved in MDSC expansion include IL-6, STAT3, STAT5, C/EBP-β, RORC1, SOCS3, BCL3, and NOTCH (12, 77). Furthermore, recent studies have shown that in breast cancer tumor mice on a high-fat diet, the disrupted intestinal microbiota releases significant amounts of leucine, activating the mTORC1 signaling pathway in granulocyte–macrophage progenitors (GMPs) and promoting the differentiation of PMN–MDSCs (78). The mTOR signaling pathway in cancer cells drives G-CSF expression, stimulates MDSC accumulation, and promotes tumor progression (79).

The activation of MDSCs in cancer is driven by the sustained release of various signaling molecules, including IFN-γ, IL-1β, IL-4, IL-6, IL-13, TNF-α, S100A8/A9, and HMGB1 (12). Furthermore, studies utilizing mouse tumor models have demonstrated that the IRE1α and ATF6 pathways of endoplasmic reticulum stress influence the suppressive activity of PMN-MDSCs (66, 80). Endoplasmic reticulum stress is recognized as a critical driver of the pathological activation of the immunosuppressive phenotype of MDSCs (66). Notably, the activation of MDSCs may not be entirely confined to the TME, as CD84^hi^ MDSCs in the tumors of tumor-bearing mice exhibited T-cell inhibitory capabilities, while CD84^hi^ MDSCs in the spleen also significantly inhibited T-cell proliferation, suggesting pathological activation of MDSCs during their migration to the spleen (71).

### 3.3 Immunosuppressive mechanisms

MDSCs selectively engage most efficient metabolic pathways to execute their immunosuppressive functions in response to changes in the microenvironmental (81). For instance, MDSCs enhance lipid accumulation and fatty acid oxidation while reducing oxidative phosphorylation. They also increase the production of metabolites such as methylglyoxal, arginine, tryptophan, and cysteine, which are conducive to tumorigenesis (66). MDSCs regulate T-cell function by depleting essential metabolites from the TME. They express inducible NO synthase (iNOS) and Arg-1 to modulate arginine metabolism, leading to the depletion of L-arginine in the TME, suppress CD3-ζ expression in T cells, and induction of apoptosis (12–14). Effector T cells are particularly vulnerable to tryptophan depletion. MDSCs contribute to the inhibition of effector T-cell proliferation by upregulating the tryptophan metabolic enzyme indoleamine 2,3-dioxygenase (IDO) (15). In contrast, the Foxp3^+^Treg population can thrive in low-tryptophan conditions (15). Furthermore, MDSCs impair cysteine export, which limits cysteine availability in the TME, thereby affecting the metabolic requirements of CD8^+^ T-cell and inhibiting their function (15, 82). MDSCs release reactive ROS and NO in the TME, potentially forming PNT and nitrate T-cell receptor (TCR)-CD8 complexes that obstruct antigen-specific T-cell responses and influence T-cell migration (14, 16). Exosomes containing G protein-coupled receptor 84 (GPR84) secreted by MDSCs are internalized by CD8^+^ T cells, resulting in GPR84-induced senescence of these T cells via the p53 pathway (83) (**Figure 2b**).

The expression of specific molecules on the surface of MDSCs is significantly correlated with their immunosuppressive functions. Under hypoxic conditions, MDSCs upregulate the V-domain Ig suppressor of T-cell activation (VISTA) on their surface; targeting VISTA can mitigate MDSC-mediated T-cell suppression (84). The blockade of exportin 1 (XPO1) inhibits the ERK1/2-mediated MAPK pathway during MDSC differentiation, significantly reducing their inhibitory effects (85). Knockout of Ankrd22 increases the expression of CCR2 and enhances the immunosuppressive activity of PMN-MDSCs in mice (86). Additionally, Netrin-1 interacts with adenosine receptor 2B (A2BR) on MDSCs to enhance their immunosuppressive functions, whereas the deletion of free fatty acid receptor 2 (FFAR2) attenuates the immunosuppressive activity of these cells (87, 88).

MDSCs suppress the activity of NK cells through multiple mechanisms. MDSCs induce anergy in NK cells via membrane-bound TGF-β and selectively suppress IFN-γ production from NKT cells (89). Prostaglandin E2 (PGE2) produced by M-MDSCs in melanoma patients can enhance TGF-β secretion, thereby inhibiting NK cell activity (90). In studies involving mouse models of liver cancer, MDSCs have been shown to inhibit NK cell cytotoxicity, as well as the expression of natural killer cell group 2D (NKG2D) and IFN-γ production, both *in vivo* and *in vitro* (91). Tumor-infiltrating NK cells exhibit significantly reduced expression of activating proteins, including DNAM-1 and NKP30 (92). Furthermore, MDSCs can downregulate NKp30 on NK cells, leading to functional inhibition of these cells (93). Additionally, MDSCs can express PD-L1 and secrete various soluble factors, such as iNOS, ROS, IDO, and Arg-1, all of which contribute to immune suppression and inhibit NK cells activation of (66, 94) (**Figure 2b**).

### 3.4 Interactions with other dysregulated immune regulatory cells

In addition to MDSCs, M2-type TAMs and Tregs are crucial components of the immunosuppressive TIME. The interaction between MDSCs and macrophages requires direct cell-to-cell contact; moreover, IL-10 produced by MDSCs inhibits IL-12 production by macrophages, skewing them toward a type II tumor-promoting phenotype (95). This interaction not only enhances IL-10 production by MDSCs but also contributes to their immunosuppressive effects. Furthermore, IL-10 produced by MDSCs drives the differentiation and accumulation of Tregs, exacerbating the immunosuppressive environment (96). Treg cells are frequently overactivated in various cancers to sustain immune tolerance and homeostasis, impeding effective anti-tumor immunity (97). Treg cells secrete inhibitory cytokines such as IL-10, IL-35, and TGF-β, which collaboratively regulate the expression of specific inhibitory receptors and exhaustion-related genes in tumor-infiltrating CD8^+^ T cells (98). MDSCs promote T cell immunosuppression through their ability to induce and recruit inhibitory Tregs, while the depletion of Tregs leads to a reduction in MDSC numbers, indicating a reciprocal relationship between MDSCs and Tregs (99).

## 4 CSCs and MDSCs in the TME

In the TME, cancer cells and their antagonists, namely immune cells, engage in constant interact ions that significantly influence the fate of solid tumors (100). For example, primary tumors release a variety of chemokines and cytokines that recruit immunosuppressive cell populations, thereby inhibiting the cytotoxic functions of NK cells and CD8^+^ T cells (7). These immunosuppressive cells include MDSCs, M2 macrophages, and FoxP3^+^Tregs (101). Conversely, the immunosuppressive signals generated by these cells also affect the stemness of cancer cells. In the subsequent sections, we discuss the interactions between CSCs and MDSCs (**Table 1**).

**Table 1:** Bidirectional crosstalk between MDSCs and CSCs in the TME.

Crosstalk direction	Cancer type	Cross signaling	Function	References
Effect of CSCs on MDSCs	Breast cancer	ALDH1A1 reduces intracellular pH, promoting TAK1 phosphorylation, NF-κB activation, and GM-CSF secretion	Promotes MDSC expansion; enhances MDSC-mediated immunosuppression (reduces the proportion of IFN-γ^+^/TNFα^+^ CD8^+^ T cells)	(21)
	Human glioblastoma multiforme	Paracrine signaling via chemokines (including CCL2, CCL6, CCL9); CCL2 is a key mediator	Recruits M-MDSCs to the TME; enhances MDSC-mediated immunosuppression (e.g., induces T cell exhaustion via PD-1/LAG3 upregulation)	(27)
	Lymphoma	CSC-secreted OPN and tumor-derived PGE2 act synergistically	Promotes PMN-MDSC expansion; strengthens MDSC-induced immune suppression	(102)
	Human gastric cancer	Smad4-dependent signaling in CSCs	Promotes PMN-MDSC accumulation; enhances immune suppression by inhibiting DC-T cell axis, thereby suppressing anti-tumor CD8^+^ T cell response	(103)
	Colorectal cancer	DCLK1 (a CSC marker) induces CXCL1/CXCL2 via the ERK pathway	Recruits MDSCs to the tumor site; enhances MDSC-mediated suppression of anti-tumor immunity	(22)
	Hepatocellular carcinoma	ETV1-transactivated LAPTM4B promotes CXCL8 secretion by CSCs	Drives the migration of MDSCs from the bone marrow to the tumor; increases intratumoral MDSC infiltration	(104)
	Esophageal squamous cell carcinoma	NEDD9 (a CSC marker) regulates CXCL8 via the ERK pathway	Specifically recruits PMN-MDSCs to the TME; enhances PMN-MDSC accumulation	(105)
	Breast cancer	p140Cap stabilizes the β-catenin destruction complex, inhibiting β-catenin activity and reducing G-CSF secretion by CSCs	Reduces PMN-MDSC systemic mobilization (from the bone marrow to the bloodstream and then to the spleen) and intratumoral accumulation	(106)
	Glioma	CSC-secreted MIF	Activates MDSCs; enhances MDSC-mediated immunosuppression	(107)
Effect of MDSCs on CSCs	Breast cancer	MDSCs secrete IL6 (to activate the STAT3 pathway) and produce NO (to activate the NOTCH pathway); crosstalk exists between these two pathways	Promotes/maintains BCSC properties: increases the proportion of ALDH1^+^ BCSCs, enhances sphere formation ability, and upregulates the stem cell core genes Oct3/4, Sox2, and Nanog	(20)
	Breast cancer	CCL20-modulated PMN-MDSCs secrete CXCL2, which binds to CXCR2 on cancer cells and activates NOTCH1/HEY1 pathway	Enhances cancer cell stemness: increases the proportion of ALDH^+^ BCSCs, improves in vitro sphere formation ability, and upregulates stemness-related genes (ALDH1A1, Sox9, Nanog, KLF4)	(117)
	Esophageal squamous cell carcinoma	PMN-MDSCs activate the Notch pathway, upregulating NEDD9 expression	Enhances cancer cell stemness: increases the proportion of ALDH1A3^+^/CD271^+^/CD90^+^ CSCs, enhances sphere formation ability, and improves *in vivo* tumor-initiating ability	(105)
	Endometrial cancer	Tumor-derived G-CSF induces MDSC expansion; MDSCs inhibit CD8^+^ T cell function and produce PGE2	Enhances cancer cell stemness; induces tumor-associated leukocytosis; promotes chemoresistance	(108)
	Breast cancer	NAC1-expressing MDSCs mediate the NAC1-dependent signaling pathway, regulating the CD44-JAK1-STAT3 axis	Supports cancer cell stemness: upregulates stemness markers (CD44, ALDH1), maintains self-renewal capacity, and enhances *in vivo* metastatic potential	(109)
	Epithelial ovarian cancer	MDSCs induce CSF2 expression in cancer cells, which activates the p-STAT3 pathway	Promotes the formation and accumulation of CSCs; increases the proportion of ALDH^+^ CSCs, upregulates stemness-related genes (Nanog, c-MYC, Sox2, Oct4), and enhances tumor sphere and colony formation ability	(110)
	Colorectal cancer	MDSCs secrete exosomal S100A9 to activate Nox/ROS/STAT3/NF-κB signaling pathways in colorectal cancer cells	Enhances cancer cell stemness: enhances sphere formation ability, upregulates CSC marker expression (e.g., CD44, CD133, Oct4), and promotes tumor growth and recurrence	(57)
	Epithelial ovarian cancer	MDSCs produce PGE2, which binds to EP2/EP4 on cancer cells and activates the PI3K-AKT-mTOR pathway	Enhances ovarian cancer cell stemness (increases the proportion of ALDH^high^ CSCs); upregulates PD-L1 expression on CSCs	(111)
	Multiple myeloma	PMN-MDSCs induce piRNA-823, which activates DNMT3B and promotes global DNA methylation	Enhances cancer cell stemness: increases the proportion of side population cells, promotes sphere formation, and upregulates core stemness genes (Nanog, Oct4, Sox2)	(112)
	Ovarian cancer	Tumor-associated MDSCs induce microRNA101 expression, which binds to the 3'UTR of CtBP2 to inhibit its expression	Enhances cancer stemness: increases ALDH^+^ cell proportion and sphere formation ability, upregulates core stemness genes (Nanog, Oct4/3, Sox2)	(113)

### 4.1 CSCs promote the expansion of MDSCs

The most compelling evidence indicating that CSCs can promote the expansion of MDSCs is the observation that ALDH1A1 activity in CSCs enhances the expansion of MDSCs through the secretion of GM-CSF, which undermines T-cell immunity and facilitates breast cancer progression (21). Furthermore, increased expression of CCL2 has been noted in human glioblastoma multiforme stem cells characterized by a mesenchymal-immune signature, which may contribute to the elevation of M-MDSCs in glioblastoma multiforme (27). Concurrently, upregulation of IRF8 has also been documented. In murine tumor models, IRF8 levels regulate the size of peripheral MDSC pools, particularly PMN-MDSCs, without affecting the immunosuppressive functionality of the remaining MDSCs (114). Tumor-derived factors such as GM-CSF, G-CSF, and IL-6 promote myeloid differentiation and induce myeloid precursors to differentiate into MDSCs with suppressive properties (115, 116). Additionally, tumor cell-derived osteopontin (OPN) is associated with tumor stemness and can enhance the role of PGE2 in promoting medullary MDSC expansion (102). Moreover, Smad4-deficient gastric cancer cells expand into CD133^+^cancer stem-like cells, resulting in the accumulation of PMN-MDSCs and the suppression of T-cell activity (103).

### 4.2 CSCs recruit and activate MDSCs

Immature myeloid cells are recruited and significantly enriched in tumor tissues, where they become activated and acquire the suppressive properties characteristic of MDSCs (115). The CXCL-CXCR2 axis plays a crucial role in the recruitment and trafficking of MDSCs. Doublecortin-like kinase 1 (DCLK1), a biomarker of colorectal CSCs, induces the expression of CXCL1 and CXCL2 through the ERK pathway, facilitating the recruitment of MDSCs into an immunosuppressive TME and promoting tumor growth (22). Notably, the knockout of the CSC biomarker DCLK1 leads to the elimination of colorectal cancer cells in immune-competent hosts (22). Additionally, the expression of lysosome-associated transmembrane protein 4B (LAPTM4B) in hepatocellular carcinoma activates markers associated with liver CSCs and significantly upregulates and secretes CXCL8, which in turn promotes the migration of MDSCs to tumors (104). In esophageal squamous cell carcinoma, NEDD9 regulates CXCL8 to recruit MDSCs into tumors via the ERK pathway (105). The p140Cap adapter protein negatively regulates β-catenin activity, constraining the breast CSC compartment and resulting in decreased G-CSF secretion and diminished accumulation of polymorphonuclear MDSCs (106). Glioma stem cells activate MDSCs by secreting macrophage migration inhibitory factor (MIF), thereby suppressing immune responses (107). Notably, glioma stem cells are in spatial proximity to MDSCs. Furthermore, in mice, CCR2, along with its ligands CCL2 and CCL7, is essential for the migration of MDSCs from the bloodstream to the tumor. CCL2 facilitates the recruitment of MDSCs to tumors and promotes immunosuppression in a CCR2-dependent manner, ultimately contributing to tumor growth (**Figure 2c**).

### 4.3 MDSCs maintain the stemness of cancer cells

On the other hand, MDSCs were also found to directly promote and maintain the CSC pool in breast cancer through two integrated signaling pathways—IL6/STAT3 and NO/NOTCH (20). For example, CCL20 modulates polymorphonuclear MDSCs via NOTCH1/HEY1 signaling, thereby enhancing the stemness and self-renewal capabilities of breast cancer cells through the CXCL2-CXCR2 pathway (117). In esophageal squamous cell carcinoma, MDSCs promote cancer cell stemness through NEDD9 via the Notch pathway (105). Moreover, G-CSF-induced MDSCs are implicated in the progression of tumor-related leukocytosis-positive endometrial cancer by inhibiting CD8^+^T cells and enhancing the stemness of endometrial cancer cells through the production of PGE2 (108). Additionally, elevated levels of NAC1 in MDSCs support the stemness of triple-negative breast cancer (109). MDSCs have been demonstrated to promote tumor sphere formation, cell colony formation, and the accumulation of CSCs in epithelial ovarian cancer by inducing the CSF2/p-STAT3 signaling pathway; notably, the maintenance of stemness in epithelial ovarian cancer cells by MDSCs can be effectively reversed by depleting CSF2 or using a p-STAT3 inhibitor (110). Furthermore, MDSCs increase the expression of stemness markers, such as Nanog and c-MYC, in epithelial ovarian cancer cells during coculture . Additionally, coculturing of Hepa1–6 cells with MDSCs increased the expression of stem cell-related genes and promoted the proliferation of these cells (118).

S100A9 is highly expressed in exosomes derived from PMN-MDSCs, and its blockade suppresses the stemness of colorectal cancer cells while reducing the susceptibility of mice to azoxymethane/dextran sulfate sodium-induced colitis-associated colon cancer (57). Hypoxia induces PMN-MDSCs to secrete increased amounts of exosomes in a HIF-1α-dependent manner. Conversely, respiratory hyperoxia can diminish the stemness of colorectal cancer cells by inhibiting exosome production of from PMN-MDSCs (57). PGE2 produced by MDSCs enhances the stem cell-like properties of epithelial ovarian cancer and increases the expression of tumor PD-L1. Therefore, depleting MDSCs may represent a therapeutic strategy for ovarian cancer by reducing the population of CSCs and the expression of tumor PD-L1 (111). PMN-MDSCs directly promote and maintain the stem cell-like properties of multiple myeloma stem cells, potentially through the piRNA-823 and DNA methylation pathways (112). The piRNA-PIWI axis, which is more active in undifferentiated cells than in differentiated cells, regulates epigenetic processes and is essential for stem cell renewal (119). Additionally, MDSCs inhibit T-cell activation, stimulate the expression of microRNA-101 in ovarian cancer cells by targeting the corepressor CtBP2, and promote tumor stemness (113) (**Figure 2c**).

### 4.4 The synergistic role of CSCs and MDSCs in regulatory immune responses

CSCs and MDSCs do not function independently in immune suppression; rather, they synergistically regulate the process of immune editing, thereby reinforcing the immune evasion state of tumors. CSCs diminish immunogenicity by downregulating MHC-I molecules while maintaining low levels of MHC-I to evade NK cell clearance (43). During this process, MDSCs are pre-activated by IL-6 and MIF secreted by CSCs, gradually accumulating in the TME in preparation for subsequent escape (107, 108). In this collaborative process, CSCs exacerbate the exhaustion of CD8^+^ T cells through intercellular interactions (49). The PD-L1 expressed on the surface of CSCs synergizes with PD-L1 on MDSCs, creating a microenvironment characterized by elevated PD-L1 expression (49). This dual binding accelerates the transition of CD8^+^ T cells into a terminally exhausted state, marked by epigenetic irreversibility. Consequently, restoring effector functions becomes challenging even after the removal of antigenic stimulation (48). Furthermore, TGF-β secreted by CSCs, along with IL-10 and PGE2 secreted by MDSCs, establishes a cytokine inhibitory network that collectively suppresses the proliferation and secretion of effector molecules by CD8^+^ T cells, further entrenching the immunosuppressive state (50, 95, 111). As CSCs acquire enhanced immune evasion capabilities through epigenetic editing, they secrete substantial amounts of factors such as GM-CSF, CXCL1, CXCL2, and CXCL8, which recruit MDSCs to the tumor core (21, 22). MDSCs protect CSCs through a dual mechanism: on one hand, they inhibit the cytotoxic functions of CD8^+^ T cells and NK cells by depleting arginine and tryptophan (12–14); on the other hand, they enhance the expression of PD-L1 in CSCs by secreting PGE2 and TGF-β, further undermining immune surveillance (111, 120). CSCs that are protected by MDSCs can proliferate further and produce additional immunosuppressive factors, continuously recruiting and activating MDSCs, thereby establishing a regulatory immune response loop characterized by CSC amplification, MDSC acquisition of immunosuppressive functions, and maintenance of CSC stemness. This loop has been confirmed as one of the core mechanisms underlying tumor immune therapy resistance in glioblastoma multiforme and colorectal cancer (22, 47).

## 5 CSCs and MDSCs in metastasis

The detrimental effects of the tumor on the host not only remain locally but are also transmitted far away. However, tumor metastasis is a complex and dynamic process that involves various stages, including triggering angiogenesis by tumor cells, infiltration of the surrounding stroma, entry of tumor cells into the bloodstream, and successful exit of circulating tumor cells (CTCs) from blood vessels to establish and increase within the premetastatic microenvironment of secondary sites (121, 122). The interaction between CSCs and MDSCs is involved in every step of these metastatic processes.

### 5.1 Angiogenesis

Early-stage tumors may remain dormant and avascular, relying on diffusion from surrounding tissues to obtain oxygen and nutrients (123). Once tumors exceed a few cubic millimeters in size, they continuously release or upregulate various proangiogenic factors that activate endothelial cells, pericytes, tumor-associated fibroblasts, and immune cells, thereby stimulating angiogenesis to meet the demands of tumor growth and metabolism (124). Glioma stem cells are attracted to endothelial cells through the SDF-1/CXCR4 axis and are encouraged to differentiate into pericytes primarily by TGF-β (125). CSC markers associated with breast cancer, such as CD133, ALDH1, and CD44^+^/CD24^-^, are positively correlated with angiogenic mimicry and the molecules involved in vasculogenic mimicry formation (126). CSCs are more closely associated with vasculogenic mimicry than with endothelium-dependent vessels, with tumor cells within the vasculogenic mimicry channel originating from CSCs (127). Specifically, CD133^+^cells, a subgroup of triple-negative breast CSCs, undergo transdifferentiation to acquire an endothelial cell phenotype and exhibit high expression of VE-cadherin, MMP2, and MMP9, thereby facilitating the development of vasculogenic mimicry (127).

In addition to CSCs, tumor-recruited MDSCs were also found to facilitate angiogenesis (128). The coinjection of tumor cells with tumor-derived MDSCs enhances tumor angiogenesis, as MDSCs produce MMP9, which regulates the bioavailability of vascular endothelial growth factor (VEGF) and promotes angiogenesis (129). Furthermore, MDSCs may be directly incorporated into the tumor endothelium and highly express endothelial markers such as VEGFR2 and VE-cadherin to bind VEGF, one of the most critical drivers of angiogenesis (128, 129). Coculturing tumor-derived MDSCs with endothelial cells has demonstrated that ^STAT3+^tumor-associated MDSCs induce the formation of endothelial cell tubes (130). Elevated expression of VEGF in ovarian cancer tissues promotes the aggregation of MDSCs within tumors via VEGF/VEGFR2 signaling, which inhibits the function of CTLs, promotes tumor progression, and is correlated with poor prognosis (131).

### 5.2 Epithelial–mesenchymal transition

**Figure 3:**
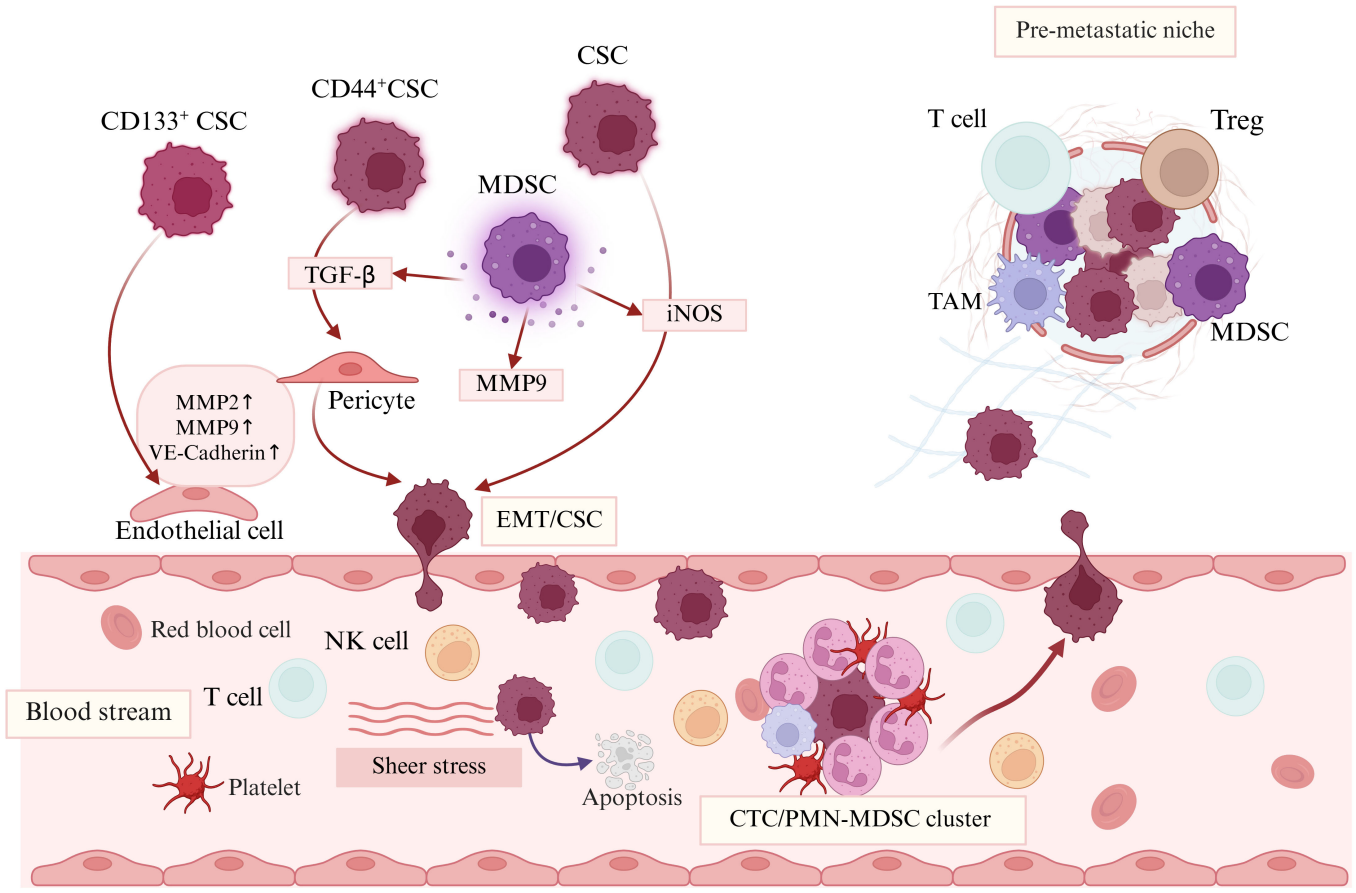
CSCs and MDSCs in tumor metastasis. CD133^+^ CSCs undergo transformation to acquire an endothelial cell phenotype, exhibiting high expression levels of VE-cadherin, MMP2, and MMP9, which facilitate the development of vasculogenic mimicry. Additionally, CSCs differentiate into pericytes in response to TGF-β stimulation. Notably, the transition of CSCs into migratory CD44^+^CSC-derived pericyte-like cells resembles EMT. Tumor-infiltrating MDSCs mediate the expression of iNOS and TGF-β, promoting the induction of EMT and the acquisition of the CSC phenotype, which in turn enhances the dissemination of tumor cells from the primary site. Furthermore, MDSCs produce MMP9, which promotes angiogenesis. Once enter bloodstream, circulating CSCs form clusters with leukocytes, primarily CTC/PMN-MDSC clusters, in response to environmental stress and significantly enhancing metastasis. After exiting the blood stream, CSCs recruit MDSCs to establish a premetastatic microenvironment, where the recruited MDSCs contribute to the improved survival of CSCs in this new environment. CTC, Circulating tumor cells; EMT, Epithelial mesenchymal transition.

Angiogenesis allows the distant travel of tumor cells, which need to be detached from their local environment first. The EMT process facilitates the detachment of cells from their epithelial counterparts, enabling them to adopt mesenchymal traits, acquire migratory capabilities, and reduce cell-to-cell adhesion. Gene mutations in cancer cells may promote a hybrid EMT phenotype; for example, this hybrid EMT state is observed in FAT atypical cadherin 1 (FAT1)-mutated human squamous cell carcinomas (132). Loss of FAT1 function activates the Ca^2+^/calmodulin-dependent protein kinase II (CAMK2)–CD44–SRC axis, promoting YAP1 nuclear translocation and zinc finger E-box binding homeobox 1 (ZEB1) expression, thereby stimulating the mesenchymal state and increasing tumor stemness and metastasis (132). Additionally, CD44^+^CSC-derived pericyte-like cells demonstrate robust transendothelial migration capabilities for intravasation and extravasation, leading to brain metastases via GPR124-mediated Wnt7-β-catenin signaling (133). Notably, the transition of CSCs into migratory CD44^+^CSC-derived pericyte-like cells resembles EMT (**Figure 3**). In contrast, the reversion of these cells into tumorigenic CSCs reflects MET, thereby supporting the idea that both EMT and MET play significant roles in metastasis (133). Furthermore, tumor-infiltrating M-MDSCs facilitate the spread of tumor cells from the primary site by inducing an EMT/CSC phenotype. Notably, M-MDSCs were detected *in situ* at the invasive edge of tumors, where strong vimentin expression was observed. The activation of the STAT1 and STAT3 signaling pathways in tumor cells, along with the mediation of iNOS expression by M-MDSCs, may contribute to the induction of the EMT/CSC phenotype (134). Additionally, M-MDSCs activate the PI3K-Akt-mTOR pathway, promoting EMT in breast cancer cells, which significantly enhances proliferation, migration, and invasion (135). MDSCs produce CCL11 to promote nonsmall cell lung cancer metastasis via the activation of the ERK and AKT signaling pathways and the induction of EMT (136). In mouse melanoma models, MDSCs are recruited to tumors through chemotactic signals from tumor-derived CXCL5. Moreover, MDSCs secrete TGF-β, epidermal growth factor, and hepatocyte growth factor, which collectively promote EMT (137). In esophageal squamous cell carcinoma, ALDH1-positive tumors are associated with aggressive tumor growth, increased IL-6, augmented EMT, and MDSC activation (138).

### 5.3 Bloodstream transmission

Following detachment from local media, intravasation and extravasation are critical processes involving the transendothelial migration of cancer cells across blood vessels. The invasion of primary CSCs into the bloodstream can occur through single-cell diffusion, collective migration, or a switching mechanism between these two modes to adapt to environmental challenges (139). This adaptation involves changes in tissue morphology and remodeling that create a migration pathway, ultimately facilitating the entry of cells into the circulatory system. A significant number of CSCs enter the bloodstream from the primary tumor daily (140) (**Figure 3**). As previously mentioned, to acquire stem cell characteristics, these cells must adapt to the changing environment from their entry into the bloodstream to the formation of metastases. When initially disengaged from the primary tumor, these circulating CSCs encounter challenges such as fluid shear and cell fragmentation within minutes of entering the bloodstream (141). Despite the threat of phagocytosis by myeloid cells, some tumor particles evade destruction and migrate into deep lung tissue, paving the way for the eventual formation of new metastatic foci (141). Furthermore, circulating CSCs experience various environmental stresses in the bloodstream, including immune responses, anoikis, oxidative stress, and a lack of oxygen and nutrition, leading to the apoptosis of most CTCs, with only a few successfully surviving to establish metastases (141, 142). Interestingly, to facilitate more effective metastasis, circulating CSCs form clusters with white blood cells (WBCs) as a clever strategy for immune evasion (**Figure 3**). In patients, CTC–WBC clusters are composed primarily of 75% myeloid cells and 25% T cells and NK cells. In contrast, mouse models exhibit an even greater proportion of myeloid cells, which account for 93% of the cluster (143). Notably, over 85% of these myeloid cells are Ly6G-positive polymorphonuclear granulocytes, with only a small number of monocytes present and no macrophages identified (143). In the context of a tumor model, myeloid-derived PMN-MDSCs are significantly amplified by tumor-derived factors. These cells can also be enriched for Ly6G positivity, suggesting that they predominantly represent PMN-MDSCs. Studies have reported the detection of CTC/PMN-MDSC clusters in patients' blood, as well as through longitudinal monitoring of animal blood, both *in vitro* and *in vivo*. Compared with those injected with either CTCs or MDSCs alone, mice injected with CTCs or PMN-MDSCs exhibited significantly enhanced dissemination and metastasis (144).

### 5.4 Premetastatic niche

The formation of a supportive metastatic microenvironment known as the premetastatic niche facilitates the establishment of tumors at distant sites after blood transmission. This niche is crucial for supporting metastatic tumor cell colonization, survival, and growth (145, 146). The infiltration of lung PMN-MDSCs enhances metastatic growth by restoring the EMT/CSC phenotype and promoting tumor cell proliferation (134). Factors such as VEGFA, which are secreted by CSCs can stimulate TAMs to produce CXCL1, which recruits CXCR2-positive MDSCs to form premetastatic niches that promote liver metastasis (147). MDSCs within these niches directly target CSCs, whereas molecules such as galectin-1 recruit PMN-MDSCs to establish a supportive environment for cancer cells. PMN-MDSCs contribute to extracellular matrix reconstruction, immunosuppression, and increased survival of CSCs in the new microenvironment (148) (**Figure 3**).

## 6 CSCs and MDSCs in targeted immunotherapies for cancer

The plasticity of CSCs and the various suppressive impacts of MDSCs on host immune surveillance outlined above constitute robust carcinogenesis. Currently, several clinical trials are focusing on key surface molecules, receptor proteins, and characteristic markers associated with these two pro-cancer cell types. Consequently, the primary obstacles to successful tumor treatment are these two mutually reinforcing cancer-driving cell types, which have emerged as major targets in clinical trials. Here, we finally evaluate the potential of current immunotherapies targeting CSCs and MDSCs, as well as the challenges that have arisen.

### 6.1 Cell-based immunotherapies

#### 6.1.1 DC vaccines

The direct delivery of CSC antigens to DCs represents a promising strategy for enhancing the efficacy of vaccination. This approach specifically targets the tumor core through a cross-presentation pathway, leading to robust cytotoxic T lymphocyte immunity. Several factors, including DC subsets, activation status, antigen loading, route of administration, and vaccination schedule, are critical in determining the success of DC vaccination (149). Data from clinical trials indicate that although DCs loaded with autologous whole tumor cell lysate antigens significantly prolong survival, the tumor-specific immune response elicited by DCs pulsed with irradiated CSCs is much more potent, leading to more significant inhibition of tumor growth (150, 151). Furthermore, immunization with a DC vaccine loaded with ALDH^+^tumor stem cell antigens following tumor resection can reduce local tumor recurrence and prolong host survival (152). Most importantly, the removal or deletion of inhibitory cells, such as MDSCs, can alleviate the immunosuppressive TME and enhance the effectiveness of CSC-DC vaccines. Research indicates that individuals who exhibited poor responses to the vaccine had significantly elevated levels of MDSCs in their peripheral blood compared with those who responded well, underscoring the influence of MDSCs on vaccine efficacy (153). These findings suggest that targeting MDSCs or selecting patients who are not immunosuppressed for prophylactic vaccination may prove advantageous.

Notably, vaccination, similar to natural tumor growth, can stimulate the expansion of MDSCs, potentially diminishing the effectiveness of the vaccine. While studies have demonstrated that vaccines containing CSC antigens can inhibit tumor growth and reduce MDSC numbers (154, 155), it is crucial to recognize that both acute and chronic infections can lead to MDSC accumulation (80). This finding suggests that vaccination itself could increase the population of MDSCs, thereby impacting the efficacy of tumor vaccines. Additionally, PMN-MDSCs inhibit the cross-presentation of DCs pulsed with CSC antigen in a nondirect cell-to-cell contact manner through lipid transfer (156). The accumulation of oxidized lipids in PMN-MDSCs is mediated by myeloperoxidase, and this lipid peroxidation may obstruct DC antigen cross-presentation via a non-cell-autonomous mechanism (156). Consequently, PMN-MDSCs can disrupt antigen cross-presentation, hindering the initiation of the cancer immune response and contributing to resistance to immunotherapy. The efficacy of DC vaccines that are solely loaded with CSC antigens is limited. Therefore, it is essential to block the crosstalk by employing a combined approach that integrates DC vaccines with the elimination of MDSCs. For example, the combination of CSC antigen DC vaccines with CXCR2 antagonists may enhance the infiltration of CD8^+^ T cells into the tumor and decrease the population of CSCs.

#### 6.1.2 CAR-T-cells

Chimeric antigen receptors (CARs) are synthetic modular proteins designed to increase the reactivity of immune cells toward specific targets. CAR-T cells can recognize CSC surface antigens, providing advantages such as sustained activity *in vitro*, specific lysis *in vivo*, and independence from MHC molecules for antigen processing. However, notably, MDSCs secrete cytokines and chemokines that diminish the effectiveness of CAR-T-cell therapy (79). Notably, MDSCs in the glioma TME express the IL-15 receptor, CAR-T cells engineered to express IL-15 create a dual-targeting system that reduces the presence of both MDSCs and glioma cells while reversing the immunosuppressive effects of MDSCs in laboratory settings (157). This strategy presents a potential approach for dual-targeted therapy using CAR-T cells within the context of an immunosuppressive TME, which typically limits their efficacy against tumors. In addition to altering the inhibitory role of MDSCs, reducing their accumulation also emerges as a viable tactic. In a mouse model of pancreatic cancer, the injection of CAR-T cells specific to pancreatic ductal adenocarcinoma-associated antigens, which coexpress CXCR4, initiated an antitumor immune response, enhanced the migration of CAR-T cells, diminished the recruitment of MDSCs, and increased the presence of CD8^+^T cells within tumor tissues (158). Since MDSCs can induce apoptosis in CD8^+^T cells and impede the cytotoxic activity of CAR-T cells, the enhanced retention of CD8^+^T cells in tumor tissues observed in mice treated with CXCR4 CAR-T cells may be attributed to the reduced recruitment of MDSCs (159).

Due to the heterogeneity of tumors and the immunosuppressive microenvironment, modifying CAR-T cells with CXCR1 or CXCR2—receptors for IL-8 released from tumors—can significantly increase the transport, migration, and persistence of T cells within tumors (160). However, notably, MDSCs also express CXCR2, which plays a role in the recruitment and trafficking of these cells. Consequently, when selecting a chemokine receptor for CAR-T-cell modification, it is crucial to consider the potential recruitment of MDSCs. Optimizing for a chemokine receptor that exclusively recruits CAR-T cells rather than inhibitory cells may represent a promising treatment strategy. Nevertheless, research on chemokine receptors in this context remains limited. Interestingly, the use of breast cancer-associated antigen-specific CAR-T cells targeting the tumor necrosis factor-related apoptosis-inducing ligand receptor (TRAIL-R2) expressed on MDSCs can induce apoptosis in MDSCs, thereby restoring their cytotoxic activity against tumor cells (161). This approach can potentially enhance the proliferation and persistence of T cells at the tumor site, ultimately aiding in the prevention of metastasis.

Clinical trials focusing on high-risk, recurrent, and refractory neuroblastoma have demonstrated that disialoganglioside (GD2)-CAR-T cells have the potential to significantly reduce tumors, elicit positive responses during early treatment stages, and decrease the presence of circulating PMN-MDSCs. Compared with nonresponders, responders presented a lower proportion of PMN-MDSCs (162). These findings suggest an inverse relationship between the number of GD2 CAR-T cells and the frequency of circulating PMN-MDSCs, indicating that PMN-MDSCs may serve as a predictive biomarker for the therapeutic response to immunotherapy. Targeting and neutralizing PMN-MDSCs with therapeutic agents could increase the antitumor activity of effector cells and protect CAR-T cells from immunosuppressive effects. In preclinical renal cancer models, the multikinase inhibitor lenvatinib has shown promise in increasing the expression of T-cell infiltration-related chemokines, reducing the frequency and immunosuppressive function of MDSCs, and improving the efficacy of CAR-T cells (163). To prevent crosstalk between CSCs and MDSCs, the development of bispecific CAR-T cells presents a viable strategy. This approach may involve chemoattractant receptor-modified CAR-T cells, such as those engineered to express CXCR4 for targeting CSC antigens. These modified cells can competitively bind to CSCs via CXCR4, thereby diminishing the secretion of chemoattractants and subsequently reducing the recruitment of MDSCs. Alternatively, dual-antigen targeting CAR-T cells could be employed, which not only target CSC antigens but also eliminate MDSCs that express the same antigens as CSCs. This strategy has the potential to reverse the immunosuppressive tumor microenvironment and enhance the efficacy of CAR-T cell therapies.

#### 6.1.3 CAR-NK-cells

NK cells have the potential to target tumor cells independently of MHC, positioning them as promising candidates for 'off-the-shelf' therapies with broad clinical applications. Compared to CAR-T cells, CAR-NK cells generally produce lower levels of IL-6, a key mediator of cytokine release syndrome (CRS) toxicity, thus diminishing the risk of severe CRS (164). CD133-CAR-NK92 cells demonstrated targeted killing of CD133-positive ovarian cancer cells, particularly when combined with cisplatin, suggesting a strategy for eliminating ovarian CSCs (165). However, MDSCs can impede NK cell cytotoxicity by suppressing the expression of NKG2D and IFN-γ (166). Interestingly, treatment with activated NK cells can decrease MDSC accumulation, facilitating the elimination of MDSCs through the NKG2D-NKG2DL axis and enhancing T-cell antitumor responses (167). CAR-NK cells engineered to target MDSC expression of NKG2D ligands in the TME by fusing the activating receptor NKG2D with the cytotoxic ζ chain of the TCR specifically target MDSCs and are less susceptible to the suppression of the TME, resulting in improved infiltration and antitumor efficacy (168).

### 6.2 Molecule-based immunotherapies

In addition to modifying effector cells to enhance antitumor immunity, current tumor immunotherapies also target critical molecules within the cells. For example, when TCRs recognize tumor antigens and initiate immune responses, their activity is meticulously regulated by immune checkpoint molecules that prevent continuous activation. CTLA-4 and PD-1 are such checkpoint molecules that serve as "brakes" on T-cell activity, whose expression can be upregulated by CSCs to apply these brakes effectively. Furthermore, IRF8, a transcription factor previously thought to be exclusively expressed in hematopoietic cells, including MDSCs, was recently found to be expressed in CSCs. Therefore, the roles of these regulatory molecules in immunotherapy and their relationships with CSCs and MDSCs are of particular interest.

#### 6.2.1 ICB

PD-1 ligation restricts immunogenic responses in T cells. Tumor-derived PD-L1 provides signals to anti-tumor PD-1^+^ T cells, thereby diminishing their functionality (169). Compared to non-CSCs, CSCs in melanoma and ovarian cancer exhibit elevated levels of PD-L1 expression (120). Silencing PD-L1 in tumor cells leads to a reduction in the CSC population. The enrichment of PD-L1 expression in CSCs is hypothesized to contribute to immune evasion in lung squamous cell carcinoma and squamous cell carcinoma of the head and neck (170, 171). Furthermore, β-catenin transcriptionally induces the N-glycosyltransferase STT3, resulting in STT3-dependent stabilization and upregulation of PD-L1 through N-glycosylation, ultimately increasing PD-L1 levels in breast CSCs (172). It is noteworthy that the relationship between tumor stemness and PD-L1 expression is not universally applicable; rather, it exhibits specific variations across different cancer types. Utilizing a logistic regression machine learning algorithm to evaluate the stemness index of cancer cells revealed a significant association between high stemness in hepatocellular carcinoma and low PD-L1 expression (173). Similarly, high stemness in glioblastoma multiforme correlated with low PD-L1 expression and a concurrent decrease in the leukocyte infiltration score within the TME (173). Furthermore, high stemness in prostate adenocarcinoma was associated with low PD-L1 expression and metastatic characteristics (173). Such tumors are less likely to respond to immune checkpoint blockade therapy due to insufficient immune cell infiltration, downregulation of pre-existing PD-L1 pathways, or the potential presence of other immune evasion mechanisms, rendering further inhibition ineffective (173). Conversely, the high expression of PD-L1 in tumor cells with a high stemness index may facilitate their immune evasion (173). In phase 3 clinical trials, the targeting and blocking of the PD-1/PD-L1 and CTLA-4 immune evasion pathways in tumor cells effectively reverses T cell exhaustion, alleviates T cell activation inhibition, and ultimately results in a significant prolongation of progression-free survival as well as an enhancement of tumor response rates, demonstrating notable antitumor effects (174, 175). This is particularly relevant for patients at high risk of immune escape, such as those with negative PD-L1 expression, as the synergistic blockade of both the PD-1 and CTLA-4 pathways can overcome the limitations associated with single-pathway treatments (174, 175).

However, some patients develop or acquire resistance to ICB therapy, underscoring the importance of elucidating the mechanisms underlying treatment variability and the need to explore additional immunotherapies that target specific immunosuppressive cells in the TME (176, 177). Notably, the frequency of circulating MDSCs is considered a negative prognostic indicator for patients receiving CTLA-4, PD-1, or PD-L1 targeted ICB therapy (177) Since, increased infiltration of MDSCs in cancer tissues is associated with resistance to various immunotherapies (178), one strategy to enhance the efficacy of ICB is to mitigate the immunosuppressive TME, with the application of inhibitors such as 1-methyl-L-tryptophan, which targets IDO, or the STAT3 antagonist JSI-124 to disrupt the immunosuppressive activity of MDSCs and optimize ICB efficacy (179). Additionally, timely inhibition of CSF-1/CSF-1R signaling can decrease the number of tumor-infiltrating MDSCs and significantly increase the effectiveness of ICB (180). Inhibiting PMN-MDSCs with specific inhibitors can enhance the therapeutic response to immune checkpoint blockade, thereby improving the control and survival of primary tumor growth in mouse models (120). S100A8/A9, expressed by MDSCs, is known to indirectly suppress CD8^+^T-cell function by upregulating Arg-1 expression in MDSCs, as well as ROS and PD-L1 expression in macrophages (181). Additionally, PGE2 produced by MDSCs enhanced the stem cell-like properties of epithelial ovarian cancer and increased tumor PD-L1 expression (111). Thus, depleting MDSCs may represent a potential strategy for treating cancer by reducing the population of CSCs and lowering tumor PD-L1 expression. The combination of MDSC-targeted drugs and ICBs has been investigated in various clinical trials, including NCT04599140. This trial evaluated the efficacy of combining nivolumab with the CXCR1/2 receptor antagonist SX-682 in patients with RAS-mutated microsatellite stable metastatic colorectal cancer (182). This combination therapy aims to increase the capacity of the immune system to target CSCs while inhibiting tumor growth and dissemination. Furthermore, pharmacological or genetic inhibition of BMI1 not only aids in eliminating BMI1^+^CSCs but also potentiates the effects of PD-1 blockade by activating the intrinsic immunity of tumor cells, thereby suppressing metastatic tumor growth and reducing the risk of tumor recurrence (183). Furthermore, combination of the molecular ICB therapy with CSC-related cellular immunotherapy could result in a synergistic efficacy that enhances the speed, depth, and persistence of clinical responses (184). For example, PD-L1-specific CAR-T cells, when used in conjunction with scFv-41BB-CD3-ζ and DC-activated T cells loaded with a CSC antigen, exhibited significant effect in killing cancer cells and reducing the tumor burden in mice, surpassing the effects of any single treatment (185).

#### 6.2.2 Transcription factor IRF8

**Figure 4:**
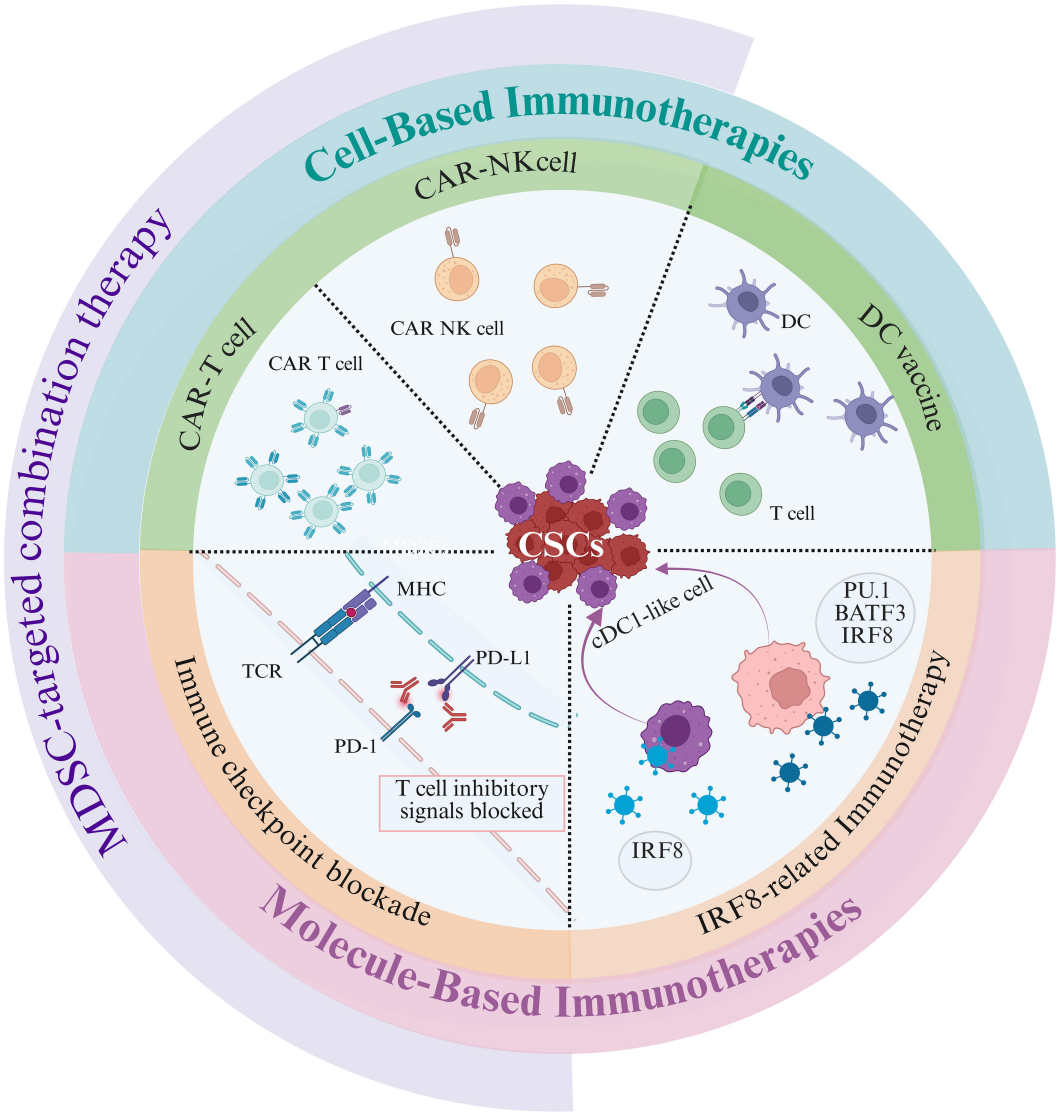
Immunotherapeutic strategies targeting CSCs and MDSCs. The immunotherapeutic strategies include cell-based immunotherapies covering DC vaccines, CAR-T or CAR-NK cells; and molecular-based treatments covering immune checkpoint blockade and IRF8-related therapies. While the cell-based approaches utilize mainly tumor immune-killing cells like T cells and NK cells to specifically target CSCs or MSDCs, reprogramming of cell differentiation via transcriptional engineering at the molecular level can directly transform MDSCs or CSCs into antigen-presenting cells to increase antitumor immunity. The combination of two strategies with ICB and CAR-T/NK therapy can lead to synergistic anti-tumor efficacy. cDC1, conventional dendritic cell type 1; CAR-T, chimeric antigen receptor T; CAR-NK, chimeric antigen receptor natural killer; GD2, disialoganglioside; IRF8, interferon regulatory factor 8; NKG2D, natural killer cell group 2D.

IRF8 functions as a negative regulator of the biology of MDSCs while also serving as a critical transcription factor for the development of type 1 conventional dendritic cells (cDC1s) (27, 186). Recent studies have utilized retroviral replication vectors to introduce IRF8 into the TME, facilitating the reprogramming of intratumoral MDSCs into cDC1-like cells (187) (**Figure 4**). This reprogramming can lead to decreased immunosuppression, enhanced antigen presentation, and an increase in infiltrating T cells within the glioblastoma multiforme TME, ultimately prolonging survival. Notably, IRF8-transduced tumor cells significantly reduce CCL2 secretion, resulting in decreased recruitment of myeloid cells into tumors via the CCL2–CCR2 axis (187). However, CSCs were also found to inappropriately 'hijack' the expression of the BM master regulatory transcription factor IRF8, which is associated with increased CCL2 expression (27). These conflicting observations have prompted investigations into the dual roles of IRF8 in glioblastoma multiforme, underscoring the urgent need to clarify its function in both CSCs and differentiated tumors. Nevertheless, targeting the activation of IRF8 to inhibit MDSC formation may represent a novel strategy for cancer immunotherapy. Additionally, IRF8^+^HLA-DR^+^cells have been identified as predictors of chemoradiotherapy-induced regression in rectal cancer patients (188).

In regenerative medicine, specific factors induce pluripotent stem cells from mouse embryonic and adult fibroblast cultures via specific factors (189). Similarly, mouse embryonic fibroblasts can be converted into cDC1-like cells through the transcription factors PU.1, IRF8, and BATF3 (190, 191). Recent studies have demonstrated that the forced expression of these transcription factors is sufficient to induce the cDC1 phenotype in 36 cell lines derived from human and mouse hematological and solid tumors (192). Within 9 days, cancer cells are reprogrammed into professional antigen-presenting cells, referred to as tumor-APCs, which acquire transcriptional and epigenetic programs, as well as functions associated with cDC1s (192). These functions include antigen phagocytosis and processing, secretion of inflammatory factors, and presentation of antigens to naive CD8^+^T cells. This reprogramming restores the expression of antigen-presenting complexes and costimulatory molecules on the tumor cell surface, thereby facilitating the presentation of endogenous tumor antigens on MHC-I and promoting targeted killing by CD8^+^T cells (**Figure 4**). Furthermore, the transcription factors PU.1, IRF8, and BATF3 were also utilized to reprogram cancer cell lines into nonadherent cultured spheroids (193). The cells reprogrammed in the tumor spheroids presented elevated levels of cDC1-associated transcription. Therefore, it is tempting to speculate that if CSCs are reprogrammed through the expression of a combination of transcription factors, including IRF8, to promote their differentiation or conversion into cell types conducive to immune-mediated killing, even if these cells are unrelated, the oncogenic potential of CSCs and the expansion of MDSCs could be diminished. Consequently, tumor eradication may become achievable, offering a promising immunotherapeutic strategy against CSCs.

### 6.3 Immunotherapeutic strategies targeting the bidirectional crosstalk between CSCs and MDSCs

Current therapeutic strategies targeting the crosstalk between CSCs and MDSCs can be categorized into four distinct classes. These strategies primarily aim to disrupt the bidirectional dependence between CSC recruitment and MDSC activation, as well as the maintenance of CSC stemness by MDSCs. The first category comprises crosstalk signal inhibitors, which either suppress the recruitment of CSCs by MDSCs (e.g., CXCR1/2 antagonists) or block the maintenance of CSC stemness by MDSCs (e.g., STAT3 inhibitors) (20, 182). The second category involves dual-cell targeted cellular therapies, where CAR-T and CAR-NK cells simultaneously recognize antigens or receptors expressed by both CSCs and MDSCs (157). The third category focuses on immune cell reprogramming, which includes inducing MDSC differentiation into antigen-presenting cells (e.g., via IRF8 transduction) and reversing CSC immune evasion (e.g., using PD-L1 inhibitors) (120, 187). Finally, the fourth category encompasses metabolic interventions, which involve inhibiting MDSC metabolic enzyme activity (e.g., Arg-1 inhibitors) and blocking metabolic support for CSCs (13).

### 6.4 The limitations and challenges of immunotherapy

CSCs represent a rare subpopulation within tumors, characterized by their self-renewal and differentiation capabilities, tumorigenicity, and contribution to tumor heterogeneity (194). These cells are often regarded as resistant to chemotherapy, radiotherapy, and immunotherapy, with treatment resistance manifesting as either intrinsic or acquired. In response to therapeutic pressure, CSCs can enter a quiescent state, allowing them to withstand DNA damage induced by radiotherapy or chemotherapy (195). Moreover, CSCs enhance their resistance to chemotherapy by eliminating ROS. They also exhibit high expression of the characteristic marker ALDH, which is closely related to multidrug resistance (196). Conversely, downregulating the CSC marker ABCG2, a member of the ABC transporter family, can increase the chemosensitivity of breast CSCs (35). Numerous markers for CSCs have been proposed across various tumor types, and advancements have been made by targeting CSC surface markers such as CD44, CD133, and EpCAM (197). However, functional differences may exist among CSC populations identified by different markers (30). The definition of a 'true' CSC population remains contentious, highlighting the need for the identification of consensus-specific markers through more rigorous experimental methodologies.

Furthermore, in response to immune attacks, CSCs employ a range of genetic and epigenetic strategies to diminish immune recognition, activate oncogenic pathways, enhance tolerance to immune-induced cytotoxicity, reduce the expression of tumor antigens, and foster the development of a protective immunosuppressive microenvironment (198). This dynamic contributes to heterogeneity among CSCs, which serves as a trap for antigenic diversity, leading to off-target effects when targeting CSCs. The mechanisms governing CSC heterogeneity are not yet fully understood, presenting challenges to the development of effective anti-CSC therapies. By simultaneously inhibiting multiple surface markers of CSCs or key signaling pathways, such as Notch, WNT, Hedgehog, and Hippo pathways, therapeutic failures caused by off-target effects of single targets can be mitigated (199). Moreover, elucidating the molecular crosstalk of key signaling pathways in CSCs is crucial for developing therapeutic strategies targeting these cells and may reveal opportunities to suppress multiple cascade reactions by directly targeting a single pathway. Additionally, beyond CAR-mediated immune redirection, bispecific or trispecific antibodies can be utilized to simultaneously recognize tumor antigens and activate effector cells. This activation occurs through binding to tumor cells, thereby activating T cells or NK cells to inhibit tumor cells that express heterogeneous antigens (200).

CSCs function as active 'architects' that facilitate interactions with other tumor components, thereby constructing a sustainable ecological niche. The complexity of tumors presents significant therapeutic challenges (201). Notably, the molecular mechanisms through which CSCs acquire drug resistance are diverse, arising from their inherent heterogeneity and intercellular communication within the tumor microenvironment. The interaction between CSCs and immunosuppressive cells leads to broader immune suppression, reshaping the stemness of tumor cells and promoting tumor formation and progression. Therefore, effective antitumor immunity requires not only functionally enhanced T cells but also the identification of pathways that regulate drug resistance, particularly those focused on non-T cell immunosuppressive cells. Customized targeted therapies aimed at the specific microenvironment of CSCs hold substantial potential for improving clinical outcomes in glioblastoma patients (202). With advancements in anticancer immunotherapies, a deeper understanding of the interactions between CSCs and the tumor immune microenvironment may be crucial for developing therapies that mitigate drug resistance tendencies, ultimately enhancing patient prognosis. It is noteworthy that this article emphasizes CSC-driven tumor progression and interactions with MDSCs. However, CSCs can further suppress immune responses by recruiting additional immunosuppressive cells beyond MDSCs, such as TAMs and Tregs, thereby facilitating the establishment of an immunosuppressive TME. Further investigation in these areas is warranted.

Furthermore, in clinical trials, actual clinical treatments targeting CSC populations are relatively scarce, highlighting significant challenges in developing effective therapies for CSCs (203). Interestingly, early clinical trial results targeting CSCs indicate that therapies aimed at CSC subpopulations may halt tumor progression without inducing tumor shrinkage, thereby leading to disease stabilization (204). Conversely, therapies focusing on the bulk tumor may result in tumor shrinkage but are less likely to achieve durable remission. Consequently, the evaluation of clinical treatments targeting CSCs may utilize progression-free survival and recurrence-free survival as assessment metrics.

## 7 Conclusions

The immune system originally evolved to combat foreign invaders and eliminate endogenous tumor cells. However, it is compromised in tumor settings, where surviving tumor cells may exhibit CSC characteristics, allowing them to continuously self-renew and differentiate while simultaneously shaping the TME via MDSCs to suppress effector T cells. Characterized by their unique capacity for self-renewal and differentiation, CSCs are fundamental drivers of tumor formation and thrive in the presence of MDSCs, which are abundant cell types in the TME that significantly inhibit the antitumor functions of T cells and NK cells. The interactions between MDSCs and CSCs are bidirectional. CSCs activate the expression of the chemokines CXCL1, CXCL2, and CXCL8 through the ERK pathway, facilitating the recruitment of MDSCs within the tumor, whereas MDSCs help maintain the stem cell characteristics of cancer cells, promote angiogenesis, and assist in CSC metastasis and colonization. Thus, these two cell types are “working partners” in driving tumor progression and immune evasion.

Targeting CSCs remains a pivotal strategy to overcome resistance to immunotherapy. Given the limitations discussed, merely blocking the crosstalk between CSCs and MDSCs is insufficient to fully dismantle the tumor immunosuppressive network. The recruitment and activation of MDSCs are fundamentally driven by CSCs, and the immunoregulatory activities of CSCs, characterized by low immunogenicity and phenotypic plasticity, constitute the root cause of resistance. Therefore, it is essential to directly target CSCs while simultaneously obstructing their bidirectional crosstalk. For instance, a combined strategy involving CD133-CAR-T cells (which eliminate CSCs), CXCR2 antagonists (which reduce MDSC recruitment), and PD-L1 inhibitors (which reverse immune checkpoints) is warranted.

Overall, the targeted elimination of CSCs while reversing immunosuppressive MDSCs represents an effective strategy for cancer treatment. Future research necessitates further explore the key molecules involved in CSC immunoregulation, such as IRF8 and CSDE1, as well as the development of dual-function drugs capable of simultaneously targeting CSC stemness and immune evasion. Additionally, employing single-cell sequencing to identify biomarkers associated with CSC-MDSC crosstalk, such as circulating PMN-MDSC/CTC clusters, is essential for implementing precision stratified therapy. Furthermore, a greater number of experimental and clinical studies are required to validate the safety and efficacy of the combined approach targeting CSCs and crosstalk blockade. Only by completely eliminating the 'driving' role of CSCs can the synergistic network with MDSCs be disrupted, thereby creating enduring opportunities for immune therapeutic responses in cancer patients.

## Glossary

A2BR: Adenosine receptor 2B
Arg-1: Arginase-1
BM: Bone marrow
CAMK2: Ca2+/calmodulin-dependent protein kinase II
CAR: Chimeric antigen receptor
cDC1: Type 1 conventional dendritic cell
CSC: Cancer stem cell
CSDE1: Cold shock domain–containing protein E1
CTC: Circulating tumor cells
CTL: Cytotoxic T lymphocyte
DAMPs: Damage-associated molecular patterns
DCLK1: Doublecortin-like kinase 1
DC: Dendritic cell
EMT: Epithelial mesenchymal transition
ER: Endoplasmic reticulum
FAT1: FAT atypical cadherin 1
FFAR2: Free fatty acid receptor 2
GD2: Disialoganglioside
GM-CSF: Granulocyte-macrophage colony-stimulating factor
GMP: Granulocyte-macrophage progenitor
GPR: G protein-coupled receptor
HIF: Hypoxia-inducible factor
ICB: Immune checkpoint blocking
IDO: Indoleamine 2,3-dioxygenase
iNOS: Inducible NO synthase
IRF: Interferon regulatory factor
LAPTM4B: Lysosomal-associated transmembrane protein 4B
LOX-1: Lectin-type oxidized LDL receptor-1
MDSC: Myeloid-derived suppressor cell
MHC: Major histocompatibility complex
MIF: Macrophage migration inhibitory factor
M-MDSC: Monocytic myeloid-derived suppressor cell
MMP: Matrix metalloproteinases
MPO: Myeloperoxidase
NE: Neutrophil elastase
NK: Natural killer
NKG2D: Natural killer cell group 2D
NO: Nitric oxide
OPN: Osteopontin
PAMPs: Pathogen-associated molecular patterns
PGE2: Prostaglandin E2
PMN-MDSC: Polymorphonuclear myeloid-derived suppressor cell
PNT: Peroxynitrite
PTGER2: Prostaglandin E receptor 2
ROS: Reactive oxygen species
SMYD3: SET and MYN domain–containing 3
TAM: Tumor-associated macrophage
TCPTP: T-cell protein tyrosine phosphatase
TCR: T-cell receptor
TME: Tumor microenvironment
TRAIL-R2: Tumor necrosis factor related apoptosis inducing ligand receptor 2
Treg: Regulatory T-cell
VEGF: Vascular endothelial growth factor
VISTA: V-domain Ig suppressor of T-cell activation
WBC: White blood cells
XPO1: Exportin 1
ZEB1: Zinc Finger E-box binding homeobox 1
